# Measurement of Photoreceptor Layer in Glaucoma: A Spectral-Domain Optical Coherence Tomography Study

**DOI:** 10.1155/2011/264803

**Published:** 2011-08-10

**Authors:** Ning Fan, Nina Huang, Dennis Shun Chiu Lam, Christopher Kai-shun Leung

**Affiliations:** ^1^Department of Ophthalmology and Visual Sciences, Hong Kong Eye Hospital, The Chinese University of Hong Kong, 147K Argyle Street, Kowloon, Hong Kong; ^2^Shenzhen Eye Hospital, Jinan University, Shenzhen 518001, China

## Abstract

*Objective.* To measure and compare photoreceptor layer thickness between normal and glaucomatous eyes using spectral-domain optical coherence tomography (OCT). *Methods*. Thirty-eight healthy normal volunteers and 47 glaucoma patients were included in the analysis. One eye from each participant was randomly selected for macula imaging by a spectral-domain OCT (3D OCT-1000, Topcon, Tokyo, Japan). The foveal and parafoveal (1.5 mm from the fovea) outer nuclear layer (ONL) and inner and outer segments (IS+OS) layer thicknesses were measured by a single masked observer. The measurements were repeated 3 times in a random sample of 30 normal eyes to determine the repeatability coefficient and intraclass correlation coefficient. *Results.* The measurement variabilities of photoreceptor thickness were low. The respective intraclass correlation coefficients of ONL and IS+OS thicknesses were 0.96 (95% confidence interval: 0.94–0.98) and 0.82 (95% confidence interval 0.70–0.90). While there were no differences in parafoveal ONL and IS+OS thicknesses between normal and glaucoma groups (*P* ≤ .410), the foveal ONL thickness was greater in glaucomatous eyes (*P* = .011) than in normal eyes. *Conclusions.* Glaucomatous damage may involve structural change in the photoreceptor layer.

## 1. Introduction

Glaucoma is characterized by progressive loss of retinal ganglion cells. However, it remains controversial whether the photoreceptor layer is involved. Kendell et al. examined the number and density of photoreceptors in postmortem eyes and found no significant difference between glaucoma and age-matched control groups [1]. By contrast, Panda and Jonas showed that the photoreceptor count was significantly lower in enucleated eyes with secondary angle-closure glaucoma [2]. In the study by Nork et al., they observed swelling of red- and green-sensitive cones in deceased donors who had a clinical diagnosis of chronic glaucoma [3]. In these studies, measurements of photoreceptors were performed in histological sections. Tissue autolysis before fixation and tissue processing may disrupt the density and architecture of the photoreceptor layer rendering assessment of the photoreceptors inaccurate. 

Optical coherence tomography (OCT) is an imaging technology that allows noninvasive in vivo measurement of the retinal layers. While the resolution of time-domain OCT has been largely limited to measuring the retinal and retinal nerve fiber layer (RNFL) thicknesses, the recent availability of spectral-domain OCT has permitted visualization of multiple intraretinal layers [4, 5]. With an axial resolution of approximately 5 *μ*m, the spectral-domain OCT distinctively discriminates the outer plexiform layer, the outer nuclear layer, the external limiting membrane, the junction of inner and outer segments of the photoreceptors, and the retinal pigment epithelium. In vivo measurement of photoreceptor layer thickness is thus possible. Investigating the involvement of photoreceptor is pertinent to understanding the extent of retinal damage in glaucoma patients and developing new psychophysical tests for glaucoma detection. The objective of this study was to measure and compare the photoreceptor layer thickness between normal and glaucomatous eyes. 

## 2. Methods

### 2.1. Subjects

Thirty-eight healthy normal Chinese volunteers and 47 glaucoma patients were consecutively recruited. They underwent a full ophthalmic examination including visual acuity, refraction, intraocular pressure measurement, and gonioscopy and fundus examination. All subjects had visual acuity of at least 20/40, spherical error within the range between +3.0 and −6.0 diopters. Subjects with clinical evidence of macular edema, retinal disease, previous refractive or retinal surgery, neurological disease, or diabetes were excluded. Normal subjects had no visual field defect, no structural optic disc abnormalities, no history of intraocular pressure >21 mmHg, and no history of ocular or neurological diseases. Glaucoma patients were defined based on the presence of visual field defects (see below) and glaucomatous optic disc changes including narrowing of neuroretinal rim and/or retinal nerve fiber layer (RNFL) defect. Only one eye was randomly selected from each subject for analysis. The study was conducted in accordance with the ethical standards stated in the 1964 Declaration of Helsinki with approval obtained from the local ethics committee. Informed consent was obtained. 

### 2.2. Measurement of Photoreceptor Layer Thickness

Spectral-domain OCT imaging was performed with the 3D OCT-1000 (Topcon, Tokyo, Japan). The details of the principle of spectral-domain OCT have been described [6, 7]. The OCT used a superluminescent diode laser with a center wavelength of 840 nm and a bandwidth of 50 nm as a light source. The acquisition rate of the 3D OCT was 18,000 A scans per second. The transverse and axial resolutions were 20 *μ*m and 5 *μ*m, respectively. In the selected eye, the macula was imaged by 6 radial lines centered at the fovea spaced 30° apart. Each scan line was 6 mm long consisting of 2048 A scans. All images were obtained with a signal strength of at least 60 as recommended by the manufacturer. Three subjects were excluded because of the presence of drusen at the macula. 

**Figure 1:**
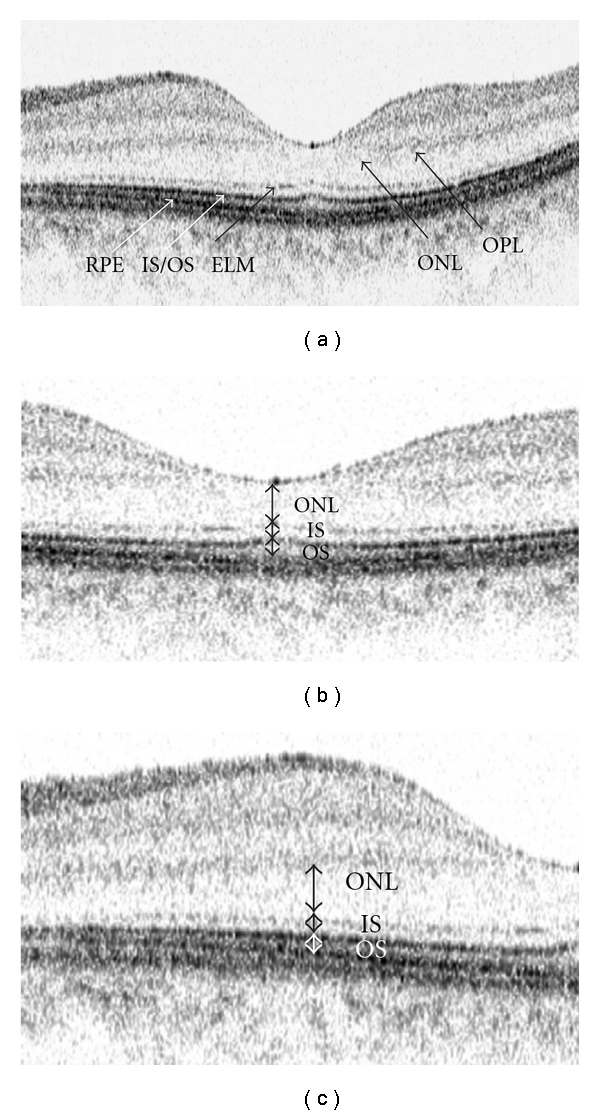
Macular imaging with spectral-domain optical coherence tomography (a). The outer nuclear layer (ONL) thickness is defined as the distance between the posterior boundary of outer plexiform layer and external limiting membrane. The inner and outer segments (IS + OS) are defined as the distance between the external limiting membrane and the RPE. Measurements were obtained at the foveola (b) and 1.5 mm from the fovea (c). OPL: outer plexiform layer; ONL: outer nuclear layer; ELM: external limiting membrane; IS + OS: inner and outer segments; RPE: retinal pigment epithelium.

Since the built-in software only provided automatic delineation and measurement of the retinal and retinal nerve fiber layer thicknesses, macular images were exported and analyzed with an image analysis software (SigmaScan Pro version 5.0; Systat software Inc., Point Richmond, Calif, USA). The outer plexiform layer, the external limiting membrane, the junction of inner and outer segments, and the retinal pigment epithelium were identified in the OCT image (Figure 1(a)). The outer nuclear layer (defined as the distance between the posterior boundary of outer plexiform layer and external limiting membrane), the inner and outer segments (the distance between the external limiting membrane and the RPE), and the total photoreceptor layer (outer nuclear layer + inner and outer segments of photoreceptors) thicknesses were manually measured in each scan. Measurements were obtained at the fovea, and at 1.5 mm away from the fovea (Figures 1(b) and 1(c)). A total of 6 images captured at different meridians were analyzed in each eye. The central foveal photoreceptor thickness was calculated by taking the average of the 6 linear scans whereas the 1.5 mm parafoveal photoreceptor thicknesses were calculated by taking the average of the 12 measurements from the 6 linear scans. All measurements were obtained by a masked observer. Thirty images from 30 normal subjects were randomly selected to determine the measurement repeatability. The foveal ONL and IS+OS layer thicknesses in each image were measured by the same observer for 3 times in 3 separate occasions. 

### 2.3. Visual Field Examination

Standard visual field testing was performed using static automated white-on-white threshold perimetry (SITA Standard 24-2, Humphrey Field Analyzer II, Carl Zeiss Meditec, Dublin, Calif, USA). A visual field was defined as reliable when fixation losses were less than 20% false positive and false negative errors were less than 15%. Visual field sensitivity was expressed in MD (mean deviation) and PSD (pattern standard deviation), as calculated by the perimetry software. A field defect was defined as having three or more significant (*P* < .05) nonedge contiguous points with at least one at the *P* < .01 level on the same side of horizontal meridian in the pattern deviation plot, classified as outside normal limits in the glaucoma hemifield test and confirmed with at least two visual field tests. None of the normal subjects had a visual field defect. 

### 2.4. Statistical Analysis

Statistical analyses were performed using SPSS version 15.0 (SPSS Inc., Chicago, Ill, USA). Foveal and parafoveal (1.5 mm) photoreceptor thicknesses were compared with independent *t*-test. The differences of photoreceptor thicknesses among normal, mild (MD >−6 dB), and moderate to advanced (MD ≤−6 dB) glaucoma groups were compared with analysis of variance with Bonferroni correction for multiple comparisons. The repeatability coefficient (2.77x within subject standard deviation (Sw)), coefficient of variation CVw (100x Sw/overall mean) and intraclass correlation coefficient (ICC) were computed. The Sw was calculated as the square root of the within-subject mean square of error (the unbiased estimator of the component of variance due to random error) in a one-way random effects model [8]. The ICC is the ratio of the intersubject component of variance to the total variance (intersubject variance + within subject variance). 

## 3. Results

The demographics and visual field MD of the normal and glaucoma groups are shown in Table 1. There was no difference in age (*P* = .35) and refraction (*P* = .17) between the groups. The visual field MD of the normal group (−0.26 ± 1.21 dB) was greater than that of the glaucoma group (−9.91 ± 8.29 dB) (*P* < .01).  Table 2 shows the intraobserver measurement repeatability of the outer nuclear layer (ONL) and the inner and outer segments (IS+OS) layer thicknesses. The intraclass correlation coefficients of the ONL and IS+OS thicknesses were 0.964 (95% confidence interval: 0.936–0.982) and 0.821 (95% confidence interval: 0.701–0.903), respectively.

For the normal eyes, the foveal ONL, IS+OS and total photoreceptor thicknesses were 96.7 ± 10.7 *μ*m, 59.3 ± 5.5 *μ*m, and 155.6 ± 12.6 *μ*m, respectively (Table 3). These measurements were smaller compared to those obtained in glaucomatous eyes (103.7 ± 13.3 *μ*m, 59.5 ± 5.5 *μ*m, and 162.9 ± 15.9 *μ*m, resp.) with significant differences found in the foveal ONL (*P* = .01) and total photoreceptor (*P* = .03) thicknesses. The parafoveal (1.5 mm) photoreceptor measurements were smaller than the foveal measurements for both normal and glaucoma subjects (all with *P* < .01). There were no detectable differences in the parafoveal (1.5 mm) photoreceptor measurements between the normal and glaucoma groups (*P* ≥ .23) (Table 4). 

The ONL thickness was significantly greater in mild glaucomatous compared with normal eyes (*P* = .02) whereas no difference was found comparing normal and moderate to advanced glaucomatous eyes (*P* = .35). The IS+OS layer thickness was comparable among the three diagnostic groups (*P* ≥ .72). 

## 4. Discussion

There are only a few histological studies investigating the involvement of photoreceptor layer in human glaucoma. The largest series was reported by Nork et al. [3]. In their study, the maculas from 128 human eyes with a diagnosis of chronic glaucoma and 90 control eyes were examined histologically. They showed that, in a subset of glaucomatous eyes, the cone nuclei at the outer portion of ONL were enlarged and the somata were swollen. Although they did not mention the severity of glaucomatous damage, their result concurs with our observation that the foveal ONL thickness was increased in a subset of patients with mild glaucoma. Cone density is the highest at the fovea (central one degree of the macula). Swollen cone perikarya could be manifested as increase in ONL thickness. In the study by Kendell, they did not find significant difference in ONL height or photoreceptor nuclei density between 9 normal and 14 glaucoma eyes [1]. However, it is notable that the photoreceptor measurements were largely based on the peripheral retina where rods dominate. Wygnanski et al. also showed that there was no cone loss in the parafoveal area (4.5 to 6 degrees eccentricity above and below the fovea) in experimental glaucoma [9]. Likewise, we did not find any significant difference in photoreceptor thicknesses between normal and glaucomatous eyes at the parafoveal (1.5 mm) region where rods outnumber cones. Panda and Jonas reported that the number of photoreceptors was reduced in 23 eyes with angle-closure glaucoma secondary to perforating corneal injuries in comparison to 14 control eyes with malignant choroidal melanoma [2]. All the eyes had high intraocular pressure resulting in painful bullous keratopathy not amendable to anti-glaucoma treatments. Loss of photoreceptors might be a result of retinal ischemia, not glaucoma. Ocular trauma per se could also result in loss of photoreceptors [10, 11]. Collectively, our finding of increased foveal, but not the parafoveal (1.5 mm), photoreceptor thickness in glaucoma is in agreement with the existing histological studies in human eyes. 

Increased photoreceptor thickness in glaucoma has been reported by Ishikawa et al. [12]. They developed a software algorithm to perform segmentation of retinal layers at the macula imaged by a time-domain OCT (Stratus OCT, Carl Zeiss Meditec, Dublin, Calif, USA). Since it was difficult to delineate the photoreceptor layer, the OCT images were preprocessed to improve the segmentation performance. They demonstrated that the outer retinal complex (comprising the ONL and the IS+OS layer) was significantly increased in glaucoma (100.4 *μ*m) compared with the normal control (93.8 *μ*m, *P* = .035). This serendipitous result, however, was attributed by potential inaccurate definition of the outer retinal complex on the OCT A-scan profile. Two recent case series, however, showed losses in cone density and thinning of the photoreceptor outer segments in patients with glaucoma [13, 14].

Two hypotheses were proposed by Nork et al. to explain the swelling of photoreceptors in glaucoma [3, 15]. In the anterograde hypothesis, reduced choroidal blood flow causes ischemia and swelling of the photoreceptors resulting in a decrease in reuptake of glutamate. The retinal ganglion cells undergo apoptosis because of glutamate overload. In the retrograde hypothesis, the photoreceptors are directly involved as a consequence of degenerating retinal ganglion cells. If this hypothesis is correct, it is expected that there would be no photoreceptors swelling in eyes with advanced glaucoma. Nork et al. considered that the anterograde hypothesis was more plausible because they found that 5 out of the 20 eyes (25%) with severe glaucoma in their study exhibited definite photoreceptor swelling [3]. Nevertheless, our in vivo measurement, which was devoid of the effect of tissue autolysis and fixation artifact, demonstrated that, while thickening of the ONL was observed in patients with mild glaucoma (visual field MD >−6 dB), the ONL thickness was comparable between the normal and the moderate to advanced glaucoma (visual field MD <−6 dB) groups. These findings align with the anterograde hypothesis. Prospective studies are needed to characterize the longitudinal profile of photoreceptor changes in glaucoma patients. 

The involvement of photoreceptors in glaucoma is supported by a number of functional studies with electroretinogram (ERG) [16–18]. In the study by Vaegan et al., they demonstrated reduction and delay of ERG a and b waves in glaucomatous eyes [16], which were comparable to those observed in early cone-rod dystrophy. Likewise, Weiner et al. showed that foveal cone ERG amplitude was subnormal in a significant proportion of glaucoma patients [17]. These studies suggest that the outer retina could be functionally abnormal in glaucoma.

In this study, the foveola was measured because this location has the highest density of cones. In fact, 80% of the retinal ganglion cells connect exclusively to cones making the foveola a strategic location studying the involvement of photoreceptors in glaucoma [19]. It is interesting to note that there were no significant differences in parafoveal photoreceptor thicknesses between the normal and glaucoma groups. This concurs with the observation that cone (but not rod) swelling is associated with glaucoma [3]. While evaluation of cellular density or morphology would be a much more sensitive approach to detect photoreceptor changes, it has not been possible to visualize individual photoreceptors with spectral-domain OCT. Nevertheless, thickness measurement could serve as a reasonable surrogate to evaluate the integrity of photoreceptors. 

In summary, in vivo measurement of photoreceptors provides a unique approach to study the association between photoreceptors and glaucoma. Although cone swelling observed in the previous histological studies offers a probable explanation for the increased foveal ONL thickness in glaucoma, infiltration of glial cells or inflammatory cells and increased extracellular matrix deposition may also contribute to the thickening. Further investigations are needed to unfold the mechanisms and functional consequences of increased photoreceptor thickness in glaucoma. 

## Figures and Tables

**Table 1:** Subject characteristics.

	Normal (*n* = 38)	Glaucoma (*n* = 47)	*P*
Gender (male/female)	13/25	27/20	.055*
Age (years) mean ± SD	53.6 ± 14.5	56.4 ± 12.9	.348^†^
Refraction (D) mean ± SD	−0.35 ± 2.65	−1.15 ± 2.65	.172^†^
Visual field MD (dB) mean ± SD	−0.26 ± 1.21	−9.91 ± 8.29	<.001^†^

D: diopters; MD: mean deviation; SD: standard deviation.

*Chi-square test.

^†^Independent sample *t*-test.

**Table 2:** Mean ± standard deviation (SD), repeatability coefficient, within-subject coefficient of variation (*CV*_*w*_), and intraclass correlation coefficient (ICC) of outer nuclear layer (ONL) and inner and outer segments (IS/OS) thicknesses in a random sample of 30 normal subjects.

	Mean ± SD (*μ*m)	Repeatability coefficient (*μ*m) (95% CI)	CV_*w*_ (%) (95% CI)	ICC (95% CI)
ONL Thickness	98.22 ± 15.47	8.470 (7.296–9.644)	3.11 (2.56–3.67)	0.964 (0.936–0.982)
IS/OS thickness	59.60 ± 7.22	8.134 (7.007–9.262)	4.93 (4.05–5.81)	0.821 (0.701–0.903)

95% CI: 95% confidence interval.

**Table 3:** Mean foveal outer nuclear layer, inner and outer segments, and photoreceptor layer thicknesses in the normal and glaucoma groups.

	ONL thickness (*μ*m)	IS/OS thickness (*μ*m)	Photoreceptor layer thickness (*μ*m)
Normal (*n* = 38)	96.7 ± 10.7	59.3 ± 5.5	155.6 ± 12.6
Glaucoma (*n* = 47)	103.7 ± 13.3	59.5 ± 5.5	162.9 ± 15.9
*P	.011	.890	.025

*Independent-sample* t*-test.

**Table 4:** Mean parafoveal (1.5 mm) outer nuclear layer, inner and outer segments and photoreceptor layer thicknesses in the normal and glaucoma groups.

	ONL thickness (*μ*m)	IS/OS thickness (*μ*m)	Photoreceptor layer thickness (*μ*m)
Normal (*n* = 38)	70.9 ± 14.0	45.2 ± 6.4	116.1 ± 18.7
Glaucoma (*n* = 47)	68.7 ± 10.7	43.6 ± 5.5	112.3 ± 14.4
*P	.410	.228	.295

*Independent-sample* t*-test.
